# Author Correction: Coupling Chlorin e6 to the surface of Nanoscale Gas Vesicles strongly enhances their intracellular delivery and photodynamic killing of cancer cells

**DOI:** 10.1038/s41598-020-75959-w

**Published:** 2020-10-30

**Authors:** Ann Fernando, Jean Gariépy

**Affiliations:** 1 Department of Pharmaceutical Sciences, The University of Toronto, 27 King’s College Circle, Toronto, Ontario M5S 1A1 Canada; 2 Department of Medical Biophysics, The University of Toronto, 27 King’s College Circle, Toronto, Ontario M5S 1A1 Canada; 3 Sunnybrook Research Institute, 2075 Bayview Ave, Toronto, ON M4N 3M5 Canada

Correction to: *Scientific Reports* https://doi.org/10.1038/s41598-020-59584-1, published online 18 February 2020

The original version of this Article contained an error in the title of the paper, where the word “enhances” was incorrectly given as “enhance”.

Additionally, there were errors in the legends of Figures 1–7 and Tables 1–2 where the word “Image Fitting” and its sizing information was incorrectly inserted.

These errors have now been corrected in the PDF and HTML versions of the Article, and in the accompanying Supplementary Information file.

